# Anti-tumor activity of patient-derived NK cells after cell-based immunotherapy – a case report

**DOI:** 10.1186/1479-5876-7-50

**Published:** 2009-06-23

**Authors:** Valeria Milani, Stefan Stangl, Rolf Issels, Mathias Gehrmann, Beate Wagner, Kathrin Hube, Doris Mayr, Wolfgang Hiddemann, Michael Molls, Gabriele Multhoff

**Affiliations:** 1 Department of Internal Medicine, University Medical Center Großhadern, Ludwig-Maximilians-Universität München, Germany; 2 Clinical Cooperation Group (CCG) "Tumor Therapy by Hyperthermia", Helmholtz Zentrum München, German Research Center for Environmental Health Munich, Germany; 3 Department of Radiation Oncology, Klinikum rechts der Isar, Technische Universität München, Germany; 4 Transfusion Medicine and Haemostaseology, University Medical Center Großhadern, Ludwig-Maximilians-Universität München, Germany; 5 Department of Pathology, University Medical Center Großhadern, Ludwig-Maximilians-Universität München, Germany; 6 Clinical Cooperation Group (CCG) "Pathogenesis of Acute Leukemias", Helmholtz Zentrum München, German Research for Environmental Health, Munich, Germany; 7 Clinical Cooperation Group (CCG) "Innate Immunity in Tumor Biology", Helmholtz Zentrum München, German Research Center for Environmental Health, Munich, Germany

## Abstract

**Background:**

Membrane-bound heat shock protein 70 (Hsp70) serves as a tumor-specific recognition structure for Hsp70-peptide (TKD) plus IL-2 activated NK cells. A phase I clinical trial has shown that repeated re-infusions of *ex vivo *TKD/IL-2-activated, autologous leukapheresis product is safe. This study investigated the maintenance of the cytolytic activity of NK cells against K562 cells and autologous tumor after 6 plus 3 infusions of TKD/IL-2-activated effector cells.

**Methods:**

A stable tumor cell line was generated from the resected anastomotic relapse of a patient with colon carcinoma (pT3, N2, M0, G2). Two months after surgery, the patient received the first monthly i.v. infusion of his *ex vivo *TKD/IL-2-activated peripheral blood mononuclear cells (PBMNC). After 6 infusions and a pause of 3 months, the patient received another 3 cell infusions. The phenotypic characteristics and activation status of tumor and effector cells were determined immediately before and at times after each infusion.

**Results:**

The NK cell ligands Hsp70, MICA/B, and ULBP-1,2,3 were expressed on the patient's anastomotic relapse. An increased density of activatory NK cell receptors following *ex vivo *stimulation correlated with an enhanced anti-tumoricidal activity. After 4 re-infusion cycles, the intrinsic cytolytic activity of non-stimulated PBMNC was significantly elevated and this heightened responsiveness persisted for up to 3 months after the last infusion. Another 2 re-stimulations with TKD/IL-2 restored the cytolytic activity after the therapeutic pause.

**Conclusion:**

In a patient with colon carcinoma, repeated infusions of *ex vivo *TKD/IL-2-activated PBMNC initiate an intrinsic NK cell-mediated cytolytic activity against autologous tumor cells.

## Background

Studies into the cellular basis of cancer immunosurveillance demonstrate that lymphocytes of both adaptive and innate immune compartments can prevent tumor development [1]. In contrast to normal tissues, tumors frequently express the stress protein heat shock protein 70 (Hsp70) on their plasma membrane, and this membrane-associated form of the Hsp70 molecule acts as a tumor-specific recognition structure for Hsp70-peptide activated natural killer (NK) cells expressing CD94 [2,3]. More recently, the glycosphingolipid globoyltriaosylceramide (Gb3) was shown to enable the selective anchorage of Hsp70 in plasma membranes of colorectal cancer cells [4]. The finding that Gb3 is predominantly found in cholesterol-rich microdomains (CRM) of tumor, but not of normal cells might provide an explanation for the tumor-specific Hsp70 membrane expression.

The region of the Hsp70 molecule which is exposed to the extracellular milieu of tumors has been identified as the 14-mer peptide TKDNNLLGRFELSG (TKD), and this resides in the amino acid sequence aa_450–463 _of the C-terminal domain substrate binding domain [5,6]. A combination of synthetically produced, GMP-grade Hsp70 peptide plus low dose IL-2 (TKD/IL-2) has been shown to stimulate the cytolytic and migratory capacity of CD3^-^/CD16/CD56^+ ^human [5,7] and mouse [8] NK cells. TKD/IL-2-activated cells specifically kill allogeneic, Hsp70 membrane-positive tumor cell lines *in vitro *[9]. Moreover, four repeated re-infusions of purified TKD/IL-2-activated NK cells have been shown to eradicate the primary tumor and prevent metastasis in a xenograft tumor mouse model of human pancreatic cancer [10]. Importantly, the induction of NK cell cytotoxicity is also possible when PBMNC rather than purified NK cells are incubated with TKD/IL-2 [11]. Furthermore, in the presence of other lymphocytes and antigen presenting cells (APC), the cytotoxic response against Hsp70 membrane-positive tumors has been found to be selectively mediated by NK cells (unpublished observations).

The enhanced cytolytic activity against Hsp70 surface-positive tumors is accompanied by, and correlates with an increased expression density of NK cell receptors including CD94/NKG2A/C, NKG2D and NCRs such as NKp30, NKp44, NKp46 [2,3,12]. The expression density of the C-type lectin receptor CD94 is associated with the capacity of NK cells to bind Hsp70 protein and TKD [2], and correlates with a strong lytic activity against Hsp70 membrane-positive tumor target cells.

The mechanism of lysis of Hsp70 membrane-positive tumors has been identified as being a perforin-independent, granzyme B-mediated apoptosis [13]. Previous studies have shown a high degree of correlation of the results of a 4-h ^51^chromium release assay and the granzyme B ELISPOT assay for measuring the granzyme B mediated killing of Hsp70 membrane-positive tumors by activated NK cells. These findings indicate that the granzyme B ELISPOT assay is a reliable test to determine Hsp70-reactivity in NK cells.

An Hsp70 membrane-positive phenotype acts as a negative prognostic marker for patients with lower rectal carcinomas and non-small cell lung cancer (NSCLC), and the overall survival of patients with Hsp70 membrane-positive cancer is significantly lower than that of their Hsp70 membrane-negative counterparts [14]. These findings highlight the clinical significance of determining the Hsp70 membrane status and the urgent need to treat patients with Hsp70 membrane-positive tumors. A phase I clinical study involving eleven patients with metastatic colorectal cancer and one patient with non-small cell lung cancer (NSCLC) has shown that the re-infusion of autologous, TKD/IL-2-activated leukapheresis products is feasible, safe and well-tolerated [15]. Furthermore, measurable immunological responses in the form of an enhanced expression of CD94 on NK cells and an increased NK cell cytolytic capacity against an allogeneic, Hsp70 membrane-positive colon carcinoma cell line CX+ were induced in 10 of the 12 patients [15]. In line with previous results from animal models [10], clinical responses fulfilling formal staging criteria were observed in 2 patients, who received more than 4 treatment cycles [15]. These promising immunological data encouraged us to treat a patient with an anastomotic relapse using a similar approach to that in the phase I clinical trial mentioned above. However, in this specific instance a tumor cell line could be established from a biopsy of the patient's tumor and its Hsp70 membrane-positive phenotype could be confirmed.

Herein, we report the kinetics of the anti-tumor immune responses in this patient who received a total of 9 re-infusions of *ex vivo *TKD/IL-2-activated, autologous leukapheresis products over a 12-month period and the clinical follow-up for 1 year. The kinetics of the initiation and maintenance of an *in vivo *cytolytic response against Hsp70-positive tumors within the first therapy cycles is in line with our previous findings from the phase I clinical trial. In this study an intrinsic NK cell activity was initiated only in patients who received more than 4 repeated re-infusion cycles of TKD/IL-2-activated, autologous PBMNC. This finding was determined in 5 patients with different tumor entities, stages and previous therapies. This is also the first observation that the administration of TKD/IL-2-activated PBMNC induces a sustained *in vivo *NK cell cytolytic response against the patient's own, Hsp70 membrane-positive tumor and the classical NK cell target K562 which persists for at least 2 months. Furthermore, we demonstrate that a decline in the *in vivo *NK cell activity can be restored by an additional 2 infusion cycles with TKD/IL-2-activated, autologous PBMNC. This indicates that the therapeutic intervention does not initiate an irreversible state of immune tolerance.

## Methods

### Ethics

Signed informed consent was obtained from the patient before the start of the first treatment and the clinical protocol was approved by the institutional ethical review board of the University Medical Center Großhadern.

### Patient characteristics and study setting

A 65 year-old male came to our attention in 03/05 at the time of an anastomotic relapse of a colon carcinoma which was initially diagnosed as being in stage IIIc (pT3, pN2 (5/17), M0, G2) using the recently revised American Joint Committee on Cancer (AJCC) Sixth Edition Cancer Staging System [16,17]. The primary tumor had been surgically removed in 02/03, but the patient had refused standard systemic adjuvant chemotherapy at the time of first diagnosis, having considered the "quality of life" implications and being aware of the magnitude of the incremental benefit.

**Figure 1:**
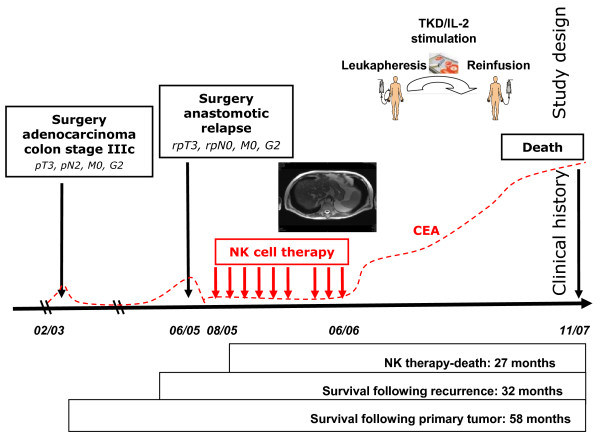
**Study design upper panel) and clinical history of the patient (bottom panel)**. A 65 year old patient with an adenocarcinoma of the colon stage IIIc pT3, N2, M0, G2 (02/03) came to our attention at the time of an anastomotic relapse in 03/05. After surgical resection of the colon carcinoma relapse in 06/05, a biopsy was provided to our laboratory for phenotypic characterization. Two months later (08/05), the NK cell therapy was started. The patient received 6 sequential leukapheresis/re-infusion cycles of autologous, *ex vivo *TKD/IL-2-activated PBMNC on a monthly basis. After a 3-month break, the patient received another 3 cell re-infusions. The patient did not show any signs of metastases at the end of the NK cell therapy, as determined by CT scan. The time interval between the beginning of the NK cell therapy and death was 27 months. Survival following recurrence and overall survival after first diagnosis was 32 and 58 months, respectively.

The patient was in a good clinical condition at the time of presentation (Karnofsky > 90%) and the resection of the anastomotic relapse three months later (06/05) revealed a high-grade colon carcinoma (rpT3, rpN0, M0, G2) (Figure 1, clinical history). Paraffin-embedded material of the primary tumor and the anastomotic relapse, as well as fresh tumor biopsy material of the anastomotic relapse, were available for laboratory use. The local tumor board recommended a post-operative systemic chemotherapy which was again refused by the patient. Although fully aware of the risk factors of his tumor disease and the recommended alternative chemotherapeutic options, the patient decided to be treated with TKD/IL-2-activated, autologous PBMNC.

In addition to the colon carcinoma the patient had a histopathologically proven highly differentiated prostate cancer which had been diagnosed in 04/02. The patient had refused resection and any pharmacological therapy of the prostate carcinoma but the prostate specific antigen (PSA) levels were determined regularly.

### *Ex vivo *stimulation of patient-derived peripheral blood mononuclear cells (PBMNC)

Two months after the surgical resection of the anastomotic relapse the experimental cell-based therapy was started in 08/05 (Figure 1, study design) after having received approval of the Institutional Ethical Committee of the Medical Faculty of the Ludwig-Maximilians-Universität Munich and the patient's written informed consent. In contrast to the phase I clinical trial, the whole procedure was repeated up to 6 times on a monthly rather than a 2-weekly basis. After a 3-month treatment pause, the patient received another 3 leukapheresis and re-infusion cycles within another 3 months. Vital and biological parameters were measured every month during the cell-based therapy and for another 12 months after the therapy had been terminated. A scheme of the therapeutic approach and the course of the disease are summarized in Figure 1.

Identical to the protocol of the clinical phase I trial [15], PBMNC concentrates were obtained by a 3–4 hour leukapheresis processing approximately 2.5 times of the patient's blood volume on a cell separator (COBE Spectra, MNC program v6.1, Heimstetten, Germany). The first leukapheresis product was aliquoted into two parts. Following erythrocyte removal by density gradient centrifugation (Ficoll-Hypaque, Life Technologies, Inc., Paisley, Scotland) in a GMP-grade closed cell culture bag and tubing system (IBM 2997 cell washer), PBMNC were counted and resuspended in GMP-grade Cellgro Stem Cell Growth Medium (CellGro SCGM, Freiburg, Germany) at a density of 10 × 10^6 ^cells/ml. The cell suspension was transferred into 250-ml Teflon cell culture bags (Vuelife, Cellgenix) and GMP-grade Hsp70-peptide TKDNNLLGRELSG (TKD, purity > 96%, lot no. 0541026; Bachem, Bubendorf, Switzerland) plus low dose IL-2 (100 IU/ml, Novartis, Nürnberg, Germany) were added.

The incubation of patient-derived PBMNC with TKD/IL-2 in an incubator (Binder, Tuttlingen, Germany) under gentle rotation (cell shaker, Binder), at 37°C in a humidified atmosphere (90%) containing 5% v/v CO_2 _for 4 days was performed to induce NK cell-mediated cytolytic activity against Hsp70 membrane-positive tumors [5]. After removal of the TKD peptide by 2 washing steps in Ringer's lactate (Braun Melsungen, Germany), cells were resuspended in 500 ml Ringer's lactate and transferred into infusion bags (600 ml, R2022, Baxter, Munich, Germany). Aliquots of the PBMNC suspension were taken for sterility tests prior to *in vitro *stimulation, on day 4 after stimulation, and directly before re-infusion.

*Ex vivo *TKD/IL-2-activated PBMNC were re-infused by intravenous (i.v.) injection within 30–60 min using an infusion set consisting of syringe and a stem cell filter (2 μm diameter, Baxter). The patient's vital parameters were monitored for 3 hours after the adoptive cell transfer.

### Clinical and laboratory follow-up

Vital and routine laboratory parameters including white blood counts, lymphocyte subpopulations, electrolytes, creatinine, urea, bilirubin, C-reactive protein, serum alkaline phosphatase, γ-glutamine transferase, alanine aminotranferease (ALT), aspartate aminotransferase (AST), lactate dehydrogenase, Quick, and aPTT were determined before each leukapheresis. Blood counts, electrolytes and coagulation tests were measured before and after each cycle of cell re-infusion. Differential blood counts and lymphocyte subpopulations were assessed in peripheral blood before each treatment cycle and in every PBMNC concentrate on the day of leukapheresis. Prostate specific antigen (PSA, Abbott, Germany) and carcinoembryonic antigen (CEA, Abbott and Elecsys/Roche, Germany) levels were determined approximately every 4 weeks during therapy and in the follow-up period.

Clinical and radiological assessments of the disease, including the proportion of the liver volume replaced by tumor (LVRT) were performed every 3 months by coloscopy, positron-emission tomography/computed tomography (PET/CT) and prostate Magnetic Resonance Imaging (MRI). Radiological responses were assessed by "Response Evaluation Criteria In Solid Tumors" (RECIST).

### Hsp70 protein and Hsp70 antibody ELISA

The concentrations of Hsp70 protein and Hsp70 antibody were measured in the patient's serum which was taken before leukapheresis L7, L8, and L9 using a sandwich ELSA kit (Duo Set IC; R&D Systems), according to the manufacturer's instructions.

### Generation of a tumor cell line

A 0.5 cm^3 ^tumor specimen from the patient's anastomotic relapse was obtained from the Department of Pathology. After washing, the tumor tissue was mechanically minced in RPMI 1640 medium supplemented with 10% v/v fetal calf serum (FCS), 1 mM sodium pyruvate, antibiotics (all from Gibco-BRL, Eggenstein, Germany) and 2 mM L-glutamine (PAN Systems, Aidenbach, Germany) and the homogenate was passed through a sterile mesh. An aliquot of the single cell suspension was immediately used for flow cytometry analysis, and the other was seeded into T-25 culture flasks in supplemented RPMI 1640 medium. After 2 weeks, adherent cells were trypsinized (trypsin/EDTA, Gibco-BRL), counted and 0.5 × 10^6 ^viable cells were resuspended in 5 ml fresh medium for further flow cytometric analyses. Aliquots of the established tumor cell line from the first 5 cell passages were stored in liquid nitrogen.

### Flow cytometric analysis of tumor and effector cells

For flow cytometry of tumor cells, 2 × 10^5 ^propidium iodide negative (viable) cells were incubated for 30 min at 4°C in the dark with the following monoclonal antibodies (mAbs): anti-fibroblast (ASO2-PE, Dianova, Hamburg, Germany), anti-MHC class I (W6/32-FITC, IgG2a; Cymbus Biotechnology, Eastleigh, UK), anti HLA-E (MEM-E/06-PE, IgG1; Biozol Diagnostica, Eching, Germany), anti-MICA/B (BAMO1, IgG1; BAMO2, IgG2a, Bamomab, Munich, Germany, kindly provided by Dr. Alexander Steinle, Tübingen), anti-ULBP-1,2,3 (AUMO2, IgG2a; BUMO1, IgG1; CUMO1, IgG1; all purchased from Bamomab), anti-human Hsp70 (cmHsp70.1-FITC, mouse IgG1, multimmune GmbH, Munich, Germany). The cmHsp70.1 mAb recognizes the sequence NLLGRFEL (aa 454–461) in the C-terminal domain of Hsp70 which is exposed to the extracellular milieu of tumor cells [5]. This sequence acts as a recognition structure for NK cells that have been stimulated either with full length Hsp70 protein or with the 14-mer Hsp70 peptide TKDNNLLGRFELSG (aa 450–463) when combined with low dose IL-2 [11,18,19]. The phenotypic characterization of the tumor was performed at the Klinikum rechts der Isar, Technische Universität München.

Unstimulated and stimulated PBMNC harvested from leukapheresis products and from the peripheral blood were incubated with the following mAbs as described above: anti-CD3 and anti-CD16/56-tricolor-conjugated (Caltag, Hamburg, Germany), anti-CD94-FITC (HP-3D9, IgG1; Becton Dickinson Pharmingen, Heidelberg, Germany) and anti-CD94-PE (Ancell Bayport, Minneapolis, MN, USA); anti-CD56-FITC (Becton Dickinson), anti-NKG2D-PE (149810, IgG1, R&D Systems, Minneapolis, MN, USA). FITC and PE labeled IgG1 and IgG2a immunoblobulins were used as isotype-matched non-specific binding controls (Caltag, Hamburg, Germany). Differential counts and determination of lymphocyte subpopulations in leukapheresis products was done with a dual-color lyse and wash method (Sumlset, BD). Flow cytometric analysis of unstimualted leukapheresis products were performed at the Klinikum rechts der Isar, Technische Universität München and at the LMU, the agreement of the results between both laboratories was verified applying Rainbow Calibration Particles (BD). Stimulated effector cells were only analyzed by flow cytometry at the Klinkum rechts der Isar, Technische Universität München.

After 2 washing steps in PBS containing 2% v/v FCS (PBS/FCS) and the addition of propidium iodide (PI, Sigma-Aldrich, Deisenhofen, Germany, stock solution 1 μg/ml), the cells were immediately analyzed by flow cytometry using a FACSCalibur™ instrument (Becton Dickinson, Heidelberg, Germany). The cell population was identified on the basis of their forward (FSC) and right angle light scatter properties (FSC vs SSC) and the fluorescence characteristics of 5,000 to 10,000 gated events were determined. Data acquisition and analysis were performed using CellQuest™ Pro software (Becton Dickinson).

### Measurement of phenotype and cytolytic activity of patient-derived PBMNC

For the *in vitro *analysis of stimulated cell populations, sterile aliquots of the leukapheresis products were incubated under identical culture conditions as the sample which was to be re-infused. The cytolytic activity of patient-derived PBMNC, without any further enrichment for NK cells, against the classical NK target cell line K562 and the autologous, Hsp70 membrane-positive tumor before and after *in vitro *stimulation with TKD/IL-2, and of freshly isolated, non-cultured patient-derived PBMNC before and after re-infusion *in vivo *was assessed using a standard 4-hour granzyme B ELISPOT assay and a ^51^chromium release assay. As the lysis of Hsp70 membrane-positive tumors by NK cells has previously been identified as being perforin-independent, granzyme B mediated apoptosis [13], this assay is suitable to determine the Hsp70-reactivity of NK cells.

For the ELISPOT assay, 96-well ELISPOT plates (Millipore GmbH, Schwalbach, Germany) were coated with capture antibody by overnight incubation at 4°C, after which they were blocked using 10% v/v FCS. The effector and target cells (3 × 10^3^) were added at different E/T ratios ranging from 20/1 to 0.5/1. After 4 hours incubation at 37°C and 2 washes, a biotinylated detecting antibody (2 μg/ml) was added. After an additional 2 washes, the presence of granzyme B was visualized using 3-amino-9-ethly-carbazole substrate solution (25 min). Spots were counted and data were analyzed using an Immuno Spot Series 3A Analyzer (CTL-Europe GmbH, Aalen, Germany).

### Antibody blocking studies

For blocking of the cytolytic activity the NK specific antibodies directed against NKp30, NKp44, NKp46 (Immunotech, Marseille, France) and the antibodies directed against Hsp70 (cmHsp70.2, multimmune GmbH) and MICA/B (BAMO1, IgG1; BAMO2, IgG2a, Bamomab, Munich, Germany) on tumor cells were used. Briefly, either effector or tumor cells were incubated with the relevant antibodies at a final concentration of 5 μg/ml for 20 min at 4°C. Then the cells were used as targets for ELISPOT assays or a standard ^51^chromium release assays, as described elsewhere [9]. Briefly, K562 and autologous tumor cells were labeled with sodium [^51^Cr] chromate (100 μCi; NEN Dupont) and used as target cells. Three thousand target cells were put into 96-well round-bottomed plates and effector cells were added at indicated E/T ratios. The cells were incubated for 4 hours at 37°C and free ^51^chromium was analyzed in a gamma counter (Coulter). % spontaneous release was both target cells was always less than 10%.

### Immunohistochemistry

For the immunohistochemical analyses, paraffin-embedded specimens were cut at 2–3 μm, using conventional histological techniques and transferred to slides (Super Frost Plus, Menzel, Germany). All staining was automatically performed on a Ventanas Benchmark^® ^XT using the following antibodies at the indicated dilutions: CD1a (Cat.1590, Immunotech, Tonsille); CD3 (SP7, NeoMarkers,1:300, Tonsille); CD4 (4B12, Novocastra,1:50, Tonsille); CD8 (C8/144B, NeoMarkers,1:50, Tonsille); CD25–305 (Novocastra,1:50, Tonsille); CD45 (LCA, 2B11+PD7, Dako, 1:1000, Tonsille); CEA (TF-3H8-1, 1:100, Ventana, Darm); CD56 (123C3.D5, 1:50, Ventana); Granzyme B (GrB-7, 1:25, Dako,); Perforin (5B10, 1:10, NeoMarkers); Hsp70 (6B3, antibody supernatant was kindly provided by Dr. Elisabeth Kremmer, Helmholtz Center Munich).

## Results and discussion

### Phenotypic characterization of patient-derived tumor

**Figure 2:**
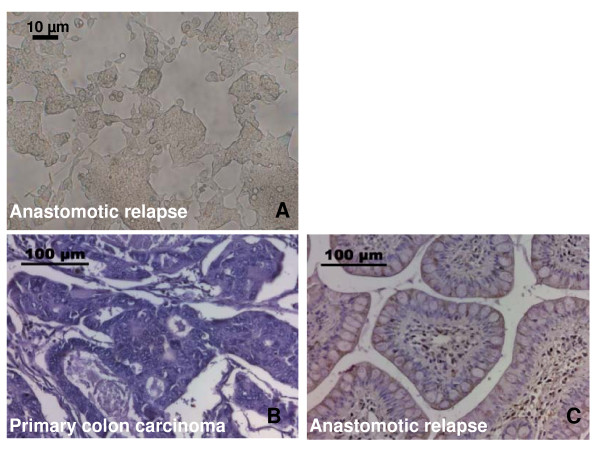
**A- Photomicrograph view of the patient-derived cell line of the anastomotic relapse**. Cells were cultured and passaged twice a week. The picture was taken at sub-confluent stage at cell passage 26; the scale bar marks 10 μm. B/C: Comparative immunohistochemical analysis of the cytosolic Hsp70 content in the primary colon carcinoma (B) and the anastomotic relapse (C). Histological slides were stained with the Hsp70 specific antibody 3B3 which reacts with Hsp70 and does not cross-react with Hsc70; the scale bar marks 100 μm.

The morphological appearance of the tumor cell line derived from the anastomotic relapse under sub-confluent culture conditions is shown in Figure 2A. Following regular twice weekly cell passages, the tumor cells formed spheroids which could be suspended by a short trypsinization step. The doubling-time of the patient-derived tumor cell line was 22 hours. The phenotype was examined on single-cell suspensions of the tumor cell line derived from the patient's tumor specimen by flow cytometry and by immunohistochemistry. The percentage of marker positive cells were determined on a minimum of six separate occasions, and the findings are summarized in Table 1. The tumor was found to be membrane MHC class I positive, but negative for the expression of HLA-E. Furthermore, the tumor revealed a strong membrane-positivity for the activatory NK cell ligands MICA/B, ULBP-3 and Hsp70. The expression of ULBP-1 and -2 was lower than that of ULBP-3. The percentage of contaminating connective tissue in the tumor cell culture, as determined using the ASO2 mAb, always remained below 5% during passages 1 to 121 (Table 1). A comparative H&E immunohistochemistry staining of the primary tumor biopsy (Figure 2B) and the anastomotic relapse (Figure 2C) revealed that the cytosolic Hsp70 content is elevated in the anastomotic relapse, thus indicating that Hsp70 levels might be associated with a more aggressive tumor stage. The antibodies directed against MICA/B and ULBP-1,2,3, which were used for flow cytometry did not stain paraffin-embedded tumor specimens (data not shown).

**Table 1:** Phenotype of the anastomotic relapse of an adenocarcinoma of the colon as determined by flow cytometry

**Cell marker**	**Positively stained cells (%)**
ASO2	2.1 ± 0.5
MHC I	89 ± 7
HLA-E	0.6 ± 1.2
MICA/B	73 ± 4.8
ULBP-1	33 ± 10
ULBP-2	64 ± 2.1
ULBP-3	98 ± 3.8
Hsp70	65 ± 1.8

### Laboratory parameters

The total number of peripheral blood leukocytes, the percentage of lymphocytes, the hemoglobin content, the number of thrombocytes, and the proportion of lymphocyte subpopulations such as CD3^+^, CD3^+^/CD4^+^and CD3^+^/CD8^+ ^T cells, CD19^+ ^B cells, CD3^+^/CD16/56^+ ^NK-like T cells, and CD3^-^/CD16/56^+ ^NK cells in the peripheral blood were within normal levels throughout the 9-month therapeutic intervention period (Table 2). The number of CD4^+^/CD25^+ ^T cells and of CD3^+^/CD16/56^+ ^NK-like T cells was always below 5%. Like in healthy human individuals the proportion of CD3^-^/CD16^+^CD56^+ ^NK cells in the peripheral blood before the start of each leukapheresis ranged between 14 to 21%. These data indicate that the adoptive transfer of *ex vivo *TKD/IL-2-activated PBMNC did not result in a significant numerical expansion or depletion of a distinct lymphocyte subpopulation.

**Table 2:** White blood counts (WBCs), hemoglobin, thrombocytes and lymphocyte subpopulations in the peripheral blood after 9 re-infusion cycles

**Cycle**	**1.**	**2.**	**3.**	**4.**	**5.**	**6.**	**7.**	**8.**	**9.**
**WBCs, hemoglobin, thrombocytes in the peripheral blood**									
[Normal range] healthy donors (n = 6)									
Leukocytes (G/l) [≥ 4]	4.1	6.3	5.0	5.0	5.2	5.2	4.0	4.9	6.5
*Lymphocytes (%) [15–40%*]	*(17)*	*(22)*	*(24)*	*(16)*	*(14)*	*(16)*	*(20)*	*(29)*	*(17)*
Hemoglobin (g/dl) [≥ 11]	13.6	14.4	14.4	12.9	12.7	12.8	13.1	12.8	11.6
Thrombocytes (G/l) [≥ 100]	146	187	135	128	149	130	157	173	177
**Lymphocyte subpopulations (%)**									
CD3^+ ^[55–95]	68	72	65	70	66	65	57	60	62
CD3^+^CD4^+ ^[35–65]	51	48	44	50	51	na*	na	na	na
CD3^+^CD8^+ ^[21–45]	18	18	18	17	17	na	na	na	na
CD19^+ ^[5-20]	14	18	18	16	17	15	14	21	12
*C*D3^+^CD16^+^CD56^+^	1	3	4	3	3	4	na	na	na
*C*D3^-^CD16^+^CD56^+ ^[5-35]	19	17	21	16	14	15	23	15	19

* na, not analyzed

The total number of nucleated cells and the total lymphocyte counts within the 9 leukapheresis products ranged between 1.1 × 10^10 ^to 1.7 × 10^10 ^and 4.3 × 10^9 ^to 8.5 × 10^9^, respectively (Table 3). The number of NK cells ranged from 0.9 × 10^9 ^(lowest value, 5^th ^cycle) to 1.9 × 10^9 ^(highest value, 4^th ^cycle), and this corresponded to 16% to 25% of the respective total lymphocyte population. These parameters were not significantly different to those obtained in the previous clinical phase I dose-escalating study [15]. In this study the total lymphocyte counts in the 12 cancer patients ranged from 0.7 × 10^9 ^to 8.5 × 10^9^and the number of activated NK cells ranged from 0.1 × 10^9 ^to 1.5 × 10^9^.

**Table 3:** Number of re-infused total nuclear cells, total lymphocytes and total NK cell counts

**Cycle**	**1.**	**2.**	**3.**	**4.**	**5.**	**6.**	**7.**	**8.**	**9.**
**Total nuclear cells, lymphocytes, NK cells in the leukapheresis products**									
Total nuclear cells (×10^10^)	1.1	see 1.	1.5	1.2	1.4	1.7	1.7	1.3	1.2
Total lymphocytes (×10^9^)	7.6		8.5	8.3	4.3	5.8	6.3	5.1	6.9
*Lymphocytes (%)*	*(69)*		*(57)*	*(69)*	*(31)*	*(34)*	*(37)*	*(39)*	*(58)*
Total NK cells (×10^9^)	1.8		1.3	1.9	0.9	1.4	1.4	1.2	1.7
*NK cells (%)*	*(24)*		*(16)*	*(23)*	*(20)*	*(24)*	*(23)*	*(23)*	*(25)*

In the follow-up period of approximately 1 year after termination of the cell-based therapy (06/06), which included a chemoembolisation therapy consisting of Gemcitabine (Gem), Irinotecan (Irino), Epirubicin (Epi), and Oxaliplatin (Oxa), the leukocyte and lymphocyte dropped below normal levels; hemoblobin levels and thrombocyte counts remained within the normal range (Table 4).

**Table 4:** Differential blood counts after termination of the cell-based therapy during chemoembolisation with Gemcitabine (Gem), Irinotecan (Irino), Epirubicin (Epi), Oxaliplatin (Oxa)

**Date**	**08/06**	**09/06 Gem**	**11/06 Gem**	**01/07 Irino**	**03/07 Epi**	**04/07 Epi**	**07/07 Oxa**	**09/07**	**10/07**
**WBCs, lymphocytes, hemoglobin, thrombocytes after cell-based therapy**									
[Normal range] healthy donors (n = 6)									
Leukocytes (G/l) [≥ 4]	6	7	7.6	9.9	6.4	6.3	3.4	3.3	3.0
*Lymphocytes (%) [15–40%*]	*(11)*	*(9)*	*(6)*	*(7)*	*(8)*	*(7)*	*(9)*	*(13)*	*(13)*
Hemoglobin (g/dl) [≥ 11]	11.7	11.2	12.3	12.5	11.2	10.2	10.3	11.1	11.7
Thrombocytes (G/l) [≥ 100]	232	239	255	189	357	347	126	115	145

Similar to the phase I clinical trial, no acute or sub-acute side effects occurred after 6 repeated infusion cycles [15]. Even after the 9^th ^leukapheresis/re-infusion cycle (L9), the therapy was well tolerated and the patient showed no signs of toxic side effects. Both the leukapheresis and re-infusion were performed in an out-patient setting on the patient's request. The patient reported a high quality of life throughout the cell-based therapeutic period.

**Figure 3:**
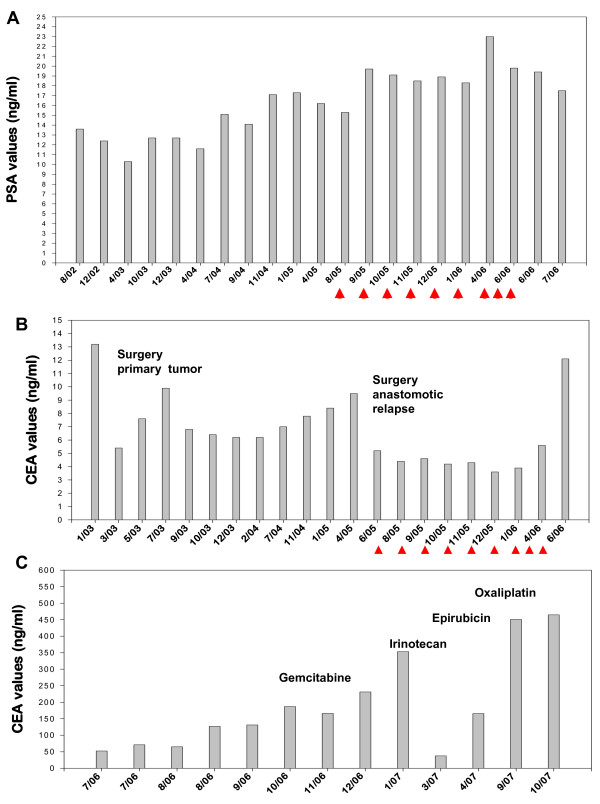
**A – Kinetics of the prostate specific antigen (PSA)**. PSA values were determined in patients's blood before, during and after adoptive transfer therapy with TKD/IL-2-activated PBMNC. The arrows indicate the time points of cell re-infusions. B – Kinetics of the carcinoembryonic antigen (CEA). CEA values were determined in patient's blood before and during the adoptive transfer therapy with TKD/IL-2-activated PBMNC. The arrows indicate the time points of cell re-infusions. In 02/03 and in 06/05 primary tumor and anastomotic relapse was surgically removed. C – Kinetics of the carcinoembryonic antigen (CEA) after completion of the cell-based therapy. CEA values were determined in patient's blood after the adoptive transfer therapy with TKD/IL-2-activated PBMNC. In 10/06 a chemoembolisation of the liver metasases with Gemcitabine, Irinotecan, Epirubicin and Oxaliplatin was initiated.

No treatment-associated changes in the standard laboratory parameters were observed during the cell-based treatment procedure (data not shown). Although levels of the tumor-associated marker PSA increased slightly from its initial value at time of first diagnosis of the prostate cancer (13.6 ng/ml) to the time point when the cell-based therapy was started (15.3 ng/ml), they remained unchanged during the cell-based therapy (Figure 3A). The level of the tumor-associated marker CEA, which was 13.2 ng/ml (01/03) before surgery of the primary colon tumor in 02/03, and 9.5 ng/ml before surgery of the anastomotic relapse in 06/05, dropped to 5.4 ng/ml after the first and to 5.2 ng/ml after the second tumor resection. During the first 6 cell re-infusions the CEA levels remained almost unaltered (L1, 4.4; L2, 4.6, L3, 4.2; L4, 4.3 L5, 3.6; L6, 3.9 ng/ml). After the 3-month break in therapy the CEA values increased to 5.6 ng/ml and after termination of the 9^th ^therapy cycle the CEA value was 12.1 ng/ml (Figure 3B).

The Hsp70 protein levels in the serum of the patient before the last three re-infusion cycles were found to be elevated compared to that measured by commercially available ELISA kits in healthy controls. Furthermore, the Hsp70 antibody levels increased more than 20-fold during the re-infusion cycles L7 and L8 and more than 10-fold during L9, as compared to that of healthy human individuals (Table 5). It remains unclear whether these findings are related to the cell-based therapy or whether these values reflect a spontaneous release of Hsp70 from tumor cells.

**Table 5:** Hsp70 protein and Hsp70 antibody levels in the serum of the patient within the last three treatment cycles, as determined by standard commercial ELISA technique

	**Hsp70 protein serum levels (ng/ml)**	**Hsp70 antibody levels (μg/ml)**
Treatment cycle*		
Before L7	10.9 ± 0.4	6,049 ± 129
Before L8	13.2 ± 0.8	5,380 ± 145
Before L9	13.3 ± 0.7	3,191 ± 122
**Healthy individuals**		
(n = 60) [40]	2.07 ± 2.74	280 ± 58
(n = 95) [41]	4.93	207 ± 55

*The data of the patient represent mean values of at least 4 independent experiments, the healthy individuals were determined with commercial ELISA kits.

### Clinical response and the patient's clinical history

Magnetic resonance imaging (MRI) of the prostate revealed that the prostate cancer remained unchanged during the adoptive transfer with TKD/IL-2-activated NK cells and the follow-up phase. The PSA levels did not significantly alter during the observation period (Figure 3A). With respect to the anastomotic relapse of the colon carcinoma, the patient remained disease-free during the first 6 cell infusion cycles, during the 3-month break in therapy and until the last cell infusion, as assessed by coloscopic analyses every 3 months, and regular whole body MRI and by PET-CT scans. These findings were in accordance with the CEA values (Figure 3B).

However, the patient developed liver metastases in both liver lobes with 20% of liver volume replaced by tumor (LVRT) 11 months after the start of the adoptive transfer of TKD/IL-2-activated effector cells and 13 months after the resection of the anastomotic relapse. At this stage a systemic chemotherapy was recommended which was refused by the patient. In the absence of any therapeutic intervention, the patient developed duodenum metastases. Four months after the last infusion cycle the CEA levels increased more than 10-fold from 12.1 (06/06) to 166.4 ng/ml (10/06) (Figure 3C). Systemic chemotherapy was further refused by the patient but in 10/06 liver lesions were treated with intra-arterial chemoembolisation consisting of Gemcitabine, Irinotecan, Epirubicin and Oxaliplatin, every 6 to 8 weeks within the following 12 months (Figure 3C). Despite a transient drop of the CEA levels from 353.4 (01/07) to 37.7 ng/ml (03/07) during the treatment with Irinotecan, the general clinical condition, liver function (cholestatic parameters), and CEA levels gradually worsened (Figure 3C), and the patient finally developed jaundice, malignant ascites and eventually died of progressive metastatic disease in 11/07.

In summary, the time interval between start of the cell-based therapy and death was 27 months. The overall survival (time interval between first diagnosis of the colon carcinoma and death) was 58 months and the survival following recurrence (time interval between anastomotic relapse and death) was 32 months. An overview of the clinical course is illustrated in the bottom panel of Figure 1.

### Immunological responses

#### NK cell phenotype and *in vitro *cytolytic activity after TKD/IL-2 stimulation

**Figure 4:**
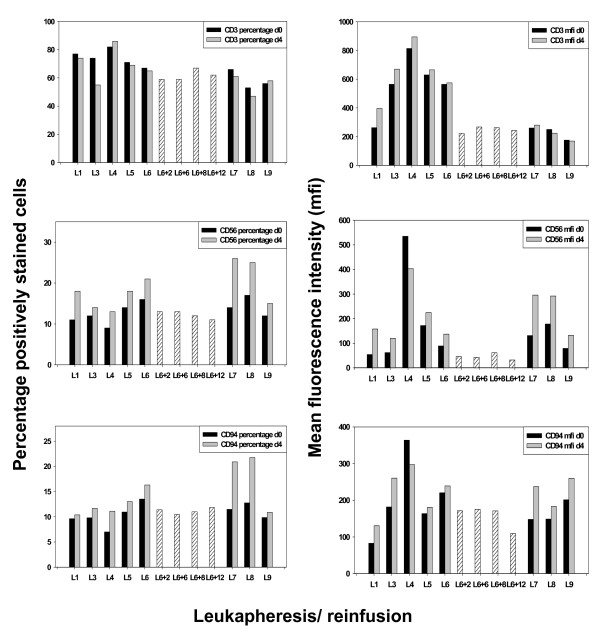
**Phenotypic changes of the effector cells before (black bars) and after (grey bars) *in vitro *TKD/IL-2 stimulation**. The percentage (left panel) and mean fluorescence intensity (mfi, right panel) values of CD3^+ ^T cells and CD3^-^/CD56^+ ^and CD3^-^/CD94^+ ^NK cells were determined before and after a 4 days *in vitro *TKD/IL-2 stimulation by flow cytometry. The hatched bars indicate T and NK cell values derived from the patients blood during the therapeutic break 2 (L6+2), 6 (L6+6), 8 (L6+8), and 12 (L6+12) weeks after re-infusion cycle L6. Only viable, propidium-iodide negative cells were gated and analyzed.

In our previous phase I study, we reported that *ex vivo *stimulation of PBMNC with TKD/IL-2 significantly increases the cytolytic activity of NK cells against Hsp70 membrane-positive tumor cell lines in 10 of 12 patients with advanced malignant disease [15]. T cells appeared not to be affected by this therapeutic approach. Furthermore, IL-2 alone had no significant effect on the cytolytic activity of PBMNC [15]. Concomitant with an increased cytotoxicity, the mean fluorescence intensity (mfi) of the NK cell receptor CD94 was found to be enhanced [15]. Here, we studied both, the percentage and the cell surface density of T and NK cell marker positive cells in the leukapheresis products before and after each of the 9 stimulation cycles of freshly isolated, non-cultured PBMNC. The percentage of CD3^+ ^T cells remained unaffected by the stimulation with TKD/IL-2 however, between leukapheresis L3 and L6 the mean fluorescence intensity (mfi) of CD3 appeared to be elevated above initial levels (Figure 4, upper right panel). Within the three months therapy break (L6+2, L6+8, L6+12 weeks after leukapheresis L6; hatched bars) the CD3 mfi values dropped down to the initial level and remained there during the last three re-infusion cycles L7–L9, on freshly isolated, non-cultured PBMNC of the patient.

With respect to the NK cell markers CD56 and the C-type lectin receptor CD94, the percentage and the mfi values were up-regulated in each treatment cycle, apart from leukapheresis L4, when a maximum in the mfi value was reached (Figure 4). The second re-infusion product was identical to the first one which was aliquoted and cryo-conserved in two parts. During the treatment pause (L6+2, L6+8, L6+12 weeks after leukapheresis L6; hatched bars) the levels of CD56 and CD94 gradually dropped but could be enhanced by additional stimulation cycles.

In summary and in line with the data of the phase I clinical trial, a comparative analysis of leukapheresis products which were obtained before and after *in vitro *stimulation with TKD/IL-2 revealed an increase in the surface densities of CD94 and CD56. This was slightly decreased after the 3-month interruption of the therapy. The subsequent 3 treatment cycles again resulted in an enhanced density of the indicated NK cell markers. Compared to unstimulated cells the density of the activatory NK cell receptors was also elevated following stimulation with TKD/IL-2. The percentage of NKG2D positively stained cells and the mean fluorescence intensity (mfi) values in the unstimulated PBMNC was 21% (39) for leukapheresis L8 and 19% (42) for L9, respectively. Following TKD/IL-2 stimulation the values increased up to 36% (52) for L8 and to 24% (45) for L9. Similarily the percentage of Natural Cytotoxicity Receptor (NCR) positively stained cells and the mfi in the TKD/IL-2-activated effector cells derived from leukapheresis 9 was elevated from 1 (21) to 3% (151) for NKp30, from 0.4 (15) to 1% (175) for NKp44, and from 2 (45) to 8% (234) for NKp46. These activation markers were only determined in leukapheresis products L8 and L9.

**Figure 5:**
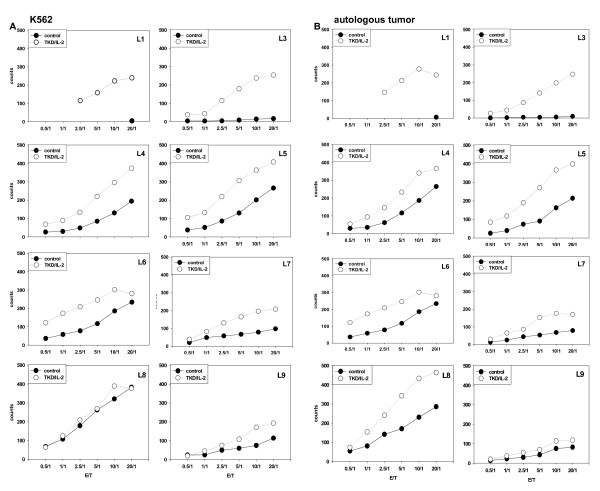
***In vitro *cytolytic activity of patient-derived PBMNC derived from L1 to L6 and L7 to L9 against K562 cells (A) and autologous tumor (B)**. The lytic activity of patient-derived PBMNC before and after stimulation with TKD/IL-2 was determined by standard granzyme B ELISPOT. Filled circles indicate the cytolytic activity of unstimulated PBMNC, open circles that of TKD/IL-2 stimulated PBMNC. Due to technical problems data from L2 are not available. Viability of the tumor target cells in each assay was > 95%.

The cytolytic activity of the patient's leukapheresis products against the classical NK cell target line K562 (Figure 5A) and against the autologous, Hsp70 membrane-positive colon carcinoma (Figure 5B) before and after TKD/IL-2 stimulation was measured by granzyme B ELISPOT assay and by ^51^chromium release assay. Before start of the therapy up to the third leukapheresis no cytolytic activity against K562 cells and autologous tumor cells was detected in patient-derived non-stimulated PBMNC (Figure 5A/B, filled circles). The cytolytic activity against both target cells could be significantly enhanced by TKD/IL-2 stimulation (Figure 5A/B, open circles). Remarkably, 1 month after re-infusion cycle 3 (before L4), freshly isolated, non-cultured PBMNC of the patient exhibited an initially increased anti-tumor activity against K562 cells (Figure 5A) and autologous tumor (Figure 5B). These effector cells also have shown a maximum in the expression density of the NK cell markers CD56 and CD94 (Figure 4).

Due to the fact that the increase in cytolytic activity following TKD/IL-2 stimulation in leukapheresis product L4 to L6 was not as pronounced as in leukapheresis L1 and L3, the therapy was stopped for 3 months. Within these 3 months the high intrinsic cytolytic activity of patient-derived PBMNC against K562 cells (Figure 5A) and autologous tumor (Figure 5B) eventually decreased but could be restored completely by two further stimulation cycles (L7, L8) with TKD/IL-2-activated leukapheresis products. In the 9^th ^stimulation cycle (L9) the *in vitro *anti-tumor activity could not be increased. The cell-based therapy was terminated at that stage.

**Figure 6:**
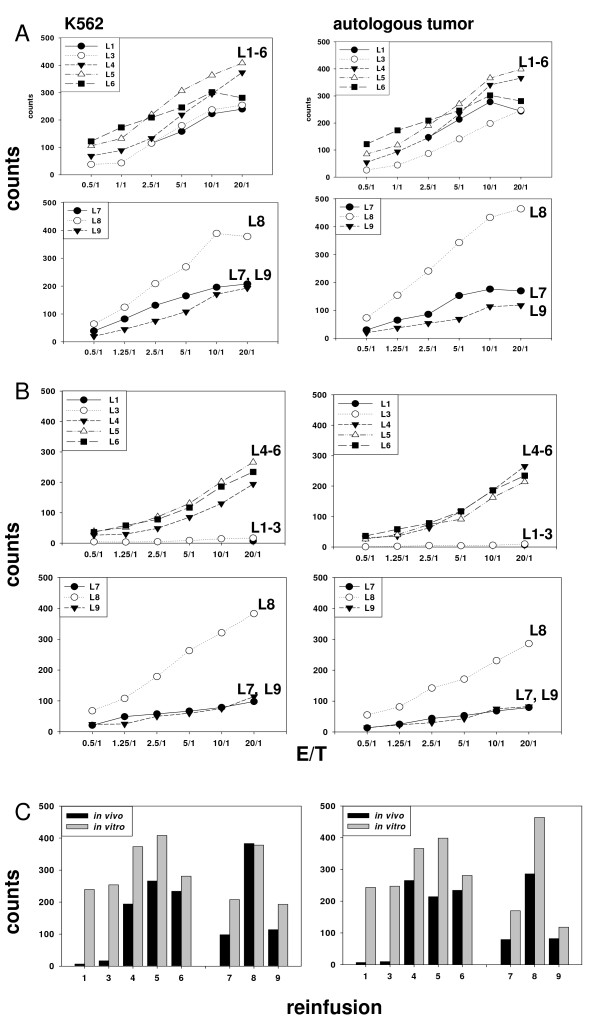
**Kinetics of the cytolytic activity of *in vitro *stimulated PBMNC (A) and freshly isolated, non-cultured PBMNC (B) of the patient derived from re-infusion cycle L1 to L6 and L7 to L9 against K562 cells (left panel) and autologous tumor (right panel)**. The lytic activity of patient-derived PBMNC was determined directly after *in vitro *stimulation (A) and 1 month after the previous cell infusion without any further *in vitro *stimulation (B) by standard granzyme B ELISPOT at E/T ratios ranging from 0.5/1 to 20/1. A direct comparison of the cytolytic activity of *in vitro *stimulated and freshly isolated, non-cultured PBMNC at the distinct E/T ratio of 20/1 is illustrated in Figure 6C.

The kinetics of the cytolytic activity of *ex vivo *stimulated PBMC derived from leukapheresis L1–L6 and L7–L9 against K562 cells (left panel) and autologous tumor (right panel) is summarized in Figure 6A. Compared to the initial level a cytotoxic response was initiated after each *ex vivo *stimulation cycle.

We have previously shown that data on the cytolytic activity of NK cells against Hsp70 membrane-positive leukemic target cells obtained using the granzyme B ELISPOT assay correlate with those obtained using a ^51^chromium release assay [20]. In line with these findings, also here the ^51^chromium release assay corroborated the granzyme B ELISPOT assay (data not shown).

#### Cytolytic activity of freshly isolated, non-cultured PBMNC after ex vivo TKD/IL-2 stimulation and adoptive transfer

The kinetics of the cytolytic response of TKD-activated NK cells within the patient was monitored by obtaining peripheral blood of the patient immediately before each cell re-infusion, 3 months after the sixth re-infusion and every 4 weeks before the re-infusion of the activated leukapheresis product L7, L8, and L9. Before start of therapy the anti-tumor activity of patient-derived PBMNC against K562 cells and Hsp70 membrane-positive autologous tumor was < 5% and remained low during the first three treatment cycles. Remarkably, one month after the third cell infusion an intrinsically increased cytolytic response against both tumor targets was firstly detected in the patients blood (Figure 6B, upper panel). This activity remained stably high during the next three re-infusion cycles (data not shown). Therapy was interrupted for 3 months after the 6^th ^re-infusion and the analysis of circulating NK cells after the therapy break revealed that the increased cytolytic capacity against K562 cells (left panel) and autologous tumor (right panel) was reduced at that time point but still elevated compared to the start of the cell-based therapy. Before stimulation cycle L8 the anti-tumor activity reached a maximum but started to decline after the 9^th ^stimulation cycle (L9, Figure 6B, lower panel).

A direct comparison of the kinetics of the cytolytic activity of the *in vitro *stimulated leukapheresis product and of freshly isolated, non-cultured PBMNC of the patient against K562 (upper panel) and autologous tumor (lower panel) during the whole therapeutic intervention (L1–L6 and L7–L9) at a distinct E/T ratio of 20/1 is summarized in Figure 6C. This kinetics of initiation and maintenance of the cytolytic response against Hsp70-positive tumors is in line with our data from the phase I clinical trial [15]. It shows that repeated re-infusions of TKD/IL-2 activated, autologous PBMNC into patients with different tumor entities, stages and previous therapies can result in NK cell activity. Moreover, this is the first observation that *ex vivo *activated NK cells can be sustained over longer periods in the blood of a patient.

Blocking studies using antibodies against activatory NK cell receptors NKp30, NKp44 and NKp46 and against the NKG2D ligand MICA/B revealed that the cytolytic response mediated by *in vitro *activated effector cells derived from leukapheresis L9 against tumor cells was not affected if compared to the effects which were mediated by isotype-matched control antibodies (data not shown). With respect to previous findings [3], we speculate that lysis of Hsp70 membrane-positive tumor cells is rather mediated through CD94/NKG2C.

### Immune reaction at the tumor site

Lymphocytes infiltrating colorectal cancers have been shown to inhibit tumor growth and their presence is associated with an improved prognosis [21,22] It has also recently been shown that the presence of infiltrating memory and effector T cells in human colorectal cancer correlates with the signs of early metastatic invasion, a less advanced pathological stage and an increased survival [23] Furthermore, Galon *et al *[24] have shown that the type, prevalence and location of immune cells within human colorectal tumors has a prognostic value which is superior to, and independent of, the histopathological methods that are currently used to stage colorectal cancer.

Based on these findings, and with the consent of the patient, paraffin-embedded specimens from the primary colon adenocarcinoma (02/03), the anastomotic relapse before start of the cell-based therapy (06/05) and a biopsy of the duodenum metastases (04/07) were analyzed by semi-quantitative immunohistochemistry. All specimens were strongly positive for the carcino-embryogenic antigen (CEA), which serves as a tumor marker for colon carcinoma. The presence of CD3^+ ^and CD45^+ ^cells was used as an indicator of T cell infiltration and the prevalence of CD4^+^, CD8^+^, CD56^+ ^cells as indicators of T helper, T cytotoxic and NK cells, respectively and CD1a was used as a marker for antigen presenting cells (APC). The expression of CD25 was considered here as a marker of lymphocyte activation since it did not show any correlation with the amount of CD4^+ ^cells which would reflect the presence of regulatory T cells (CD4^+^/CD25^+^). The expression of perforin and granzyme B provided insight into the lytic activity of infiltrating T and NK cells. In the primary tumor and in the anastomotic relapse there was a strong infiltration of CD3^+^/CD4^+ ^T cells, but no infiltration of antigen presenting cells, as determined by the marker CD1a (Table 6). The amount of T cells was lower in the metastatic tissue. In all three tumor specimen hardly any CD8^+ ^cytotoxic lymphocytes were found (Table 4). A slight increase in granzyme B-positive, CD56^+ ^NK cells was detectable in the metastatic tissue which was taken after the cell-based therapy, whereas perforin was absent (Table 4). This might be related to the fact that TKD/IL-2 activated NK cells kill their Hsp70 membrane-positive targets via a perforin-independent granzyme B mediated pathway.

**Table 6:** Semiquantitative immunohistological analysis of the tumor marker CEA, effector cell infiltration and effector cell function in the primary colon carcinoma before start of the NK cell-based therapy, the anastomotic relapse before start of the NK cell-based therapy and the duodenom metastases after finishing the NK cell-based therapy

**Cell marker**	**Primary colon tumor (02/03)**	**Anastomotic relapse (03/05)**	**Metastases (04/07)**
CEA	++++*	**++++**	**++++**
CD45 (lymphocytes)	**++**	**++**	**++**
CD3 (T cells)	**+++**	**+++**	**++**
CD4 (helper T cells)	**++**	**++**	**++**
CD8 (cytotoxic T cells)	**+/-**	**+/-**	**+/-**
CD56 (NK cells)	**-**	**-**	**+**
CD1a (APCs)	**-**	**-**	**-**
CD25 (IL-2 receptor)	**+**	**++**	**+++**
Perforin (apoptosis inducer)	**-**	**-**	**-**
Granzyme B (apoptosis inducer)	**-**	**-**	**+**

*The nomenclature means the amount of infiltrating marker-positive cells in a tumor section with a size of 2 cm^2^: -, < 50 cells; +/-, 50–80 cells; +, 80–150; ++, 150–200; +++, 200–300 cells; ++++, > 300.

## Conclusion

A previous clinical phase I trial has demonstrated that up to 6 repeated re-infusions of TKD/IL-2-activated, autologous PBMNC is safe and well-tolerated [15]. The observations that the administration of these cells induced NK cell activity against tumor cell lines expressing Hsp70 on the cell surface, as well as the unexpected clinical responses that were induced prompted additional studies. Herein, the maintenance of the cytolytic activity of *ex vivo *TKD/IL-2-activated PBMNC against a classical NK target and the autologous, Hsp70 membrane-positive tumor of a patient with an anastomotic relapse of a colon adenocarcinoma was tested. In accordance with the protocol for the clinical phase I trial, the patient received 6 cycles of *ex vivo *TKD/IL-2-activated, autologous PBMNC, that were derived form a leukapheresis, by i.v. injection. In contrast to the phase I trial protocol, the cell re-infusions were repeated every 4 instead of every 2 weeks.

No intrinsic NK cell activity was detected in patient-derived PBMNC at the beginning of the therapeutic intervention, nor was any apparent up to the third treatment cycle. However, *in vitro *incubation of PBMNC with TKD/IL-2 initiated a significant anti-tumor reactivity against the classical NK target K562 and also the autologous tumor. Most interestingly, after the fourth re-infusion cycle, patient-derived PBMNC exhibited an intrinsically enhanced NK cell activity. This finding is in line with the kinetics of the NK cell activation in patients who received more than 4 cell infusions in the phase I clinical trial [15]. Since the intervals of the cell infusions differed between the phase I clinical trial and in the present study, it is more likely to assume that the number of *ex vivo *stimulation cycles is important for the initiation of the *in vivo *immune response and not the kinetics. Phenotypic characteristics and the lytic activity against K562 cells revealed that NK cells and not T cells are responsible for the anti-tumor activity. It currently remains unclear whether this activity is due to the fact that the complete NK cell repertoire has been activated after 4 stimulation cycles or whether *ex vivo*-activated PBMNC have the capacity to activate other NK cells in the circulation of the patient. A direct stimulation of NK cells appears to be unlikely since soluble TKD-peptide was not present in the infused cell suspensions. However, it is possible that the cytolytic activity of TKD/IL-2-activated NK cells might lead to the release of cytosolic proteins [25] which enable a further secondary stimulation of NK cells *in vivo*.

Due to the fact that *in vitro *TKD/IL-2 stimulation only marginally increased the cytolytic anti-tumor activity in PBMNC obtained from the leukapheresis L4 onwards, the cell-based immunotherapy was interrupted for 3 months after the sixth re-infusion cycle. The phenotype of the pre-stimulated PBMNC that were derived from the patient's blood reflected that of the *in vitro *stimulated effector cells. Compared to the PBMNC, which were obtained before the start of the therapy, the CD3^- ^NK cells exhibited an increased density of activatory NK cell markers such as CD94/NKG2C, CD16/CD56, NKG2D, CD25 and the NCRs NKp30, NKp44, NKp46, although the absolute number of NK cells remained unaffected.

The elevated intrinsic cytolytic activity against K562 cells and autologous tumor persisted for at least 2 months and began to decline 3 months after the last cell infusion. These data might provide an insight into the life-expectancy and/or the cytolytic capacity of *ex vivo*-activated NK cells following re-infusion into a patient. Another possibility could be a numerical imbalance of active tumor-controlling NK cells and seeding tumor cells which finally results in a selection and an advantage of tumor cells with metastatic potential. Moreover, we could show that the patient's immune responses to Hsp70 membrane-positive tumors could be restored by 2 additional re-infusion cycles with TKD/IL-2-activated leukapheresis products.

A recent study from the Adjuvant Colon Cancer End Points (ACCENT) data set examined prognostic factors and survival rates following recurrence in stage II and III colon cancer in a collection of individual patient data from 18 trials testing FU-based adjuvant therapy conducted between 1978 and 1999 [26]. In this study the most important parameters were time from the initial treatment to the recurrence of disease. The median survival following recurrence was 13.1 months and was 12.5 months for patients with an initial tumor stage III. Interestingly, patients who had a recurrence following FU-based adjuvant chemotherapy had a poorer prognosis (median survival 11.5 months) than those who progressed after surgery alone (median survival 14.2 months). The patient described in the present study remained disease-free for 15 months following recurrence and died of progressive disease 32 months after diagnosis of recurrence, a time interval which is more than double that observed in the ACCENT study (32 months vs 12.5 months) [26]. Recent palliative systemic chemotherapy with newer agents has been shown to be effective and to substantially prolong survival [27-29], whereas locoregional treatments such as hepatic artery chemoembolisation currently do not provide a survival benefit for the patient [30,31]. The time interval from progression with liver lesions to death (16 months) and the overall survival (58 months) of our patient who refused systemic chemotherapy was considerably greater than that which one would expect after chemoembolisation [31], and that of stage IIIc colorectal cancer patients that undergo surgery alone (5-year median overall survival 20%) [20,32,33].

Explanations for the observed clinical outcome of the patient may be related to the patient's individual tumor disease, to the patient's immune status or to the applied cell-based therapy. The patient's anastomotic relapse did express a variety of ligands such as Hsp70, MICA-A/B, ULBP-1,2,3 which are recognized by activatory NK cell receptors. It is known that the DNA damage which is initiated in tumor cells by ionizing irradiation and certain chemotherapeutic agents elicits anti-tumor immunity [34]. These tumor cells can express "eat me" signals on their cell surface, and they can secrete/release immunostimulatory factors, such as cytokines, which in turn stimulate effector cells of the innate immune system [34].

The patient described in this report received several treatments of intra-hepatic chemoembolisation which could result in an overexpression of Hsp70 within the tumor [35-38]. As a result of tumor cell necrosis or active release Hsp70 might become available for the innate immune system [31]. Regarding these results we hypothesize that NK cells might be re-activated by stressed tumor cells.

Despite the high level of cytolytic activity over a period of 10 months, the patient died from metastatic liver disease 27 months after cell-based therapy, and 32 months after recurrence. An explanation for this might be that the tumor has escaped the control mediated by TKD/IL-2-activated NK cells *in vivo*. Furthermore, we cannot exclude that the metastases succeeded to acquire an NK cell escape mechanism such as a down-regulated activatory NK ligand expression such as Hsp70, MICA/B or ULBP-1,2,3 or an up-regulation of inhibitory NK ligands such as HLA-E molecules [23,39]. Unfortunately we are unable to address these questions due to a lack of metastatic tumor material from the patient. At the time point when metastatic disease was histologically proven, the *in vivo *cytolytic activity of patient-derived PBMNC had dropped. Interestingly, the Hsp70 antibody levels and to a lower extent also the Hsp70 protein levels in the serum were found to be highly elevated above normal levels [40,41] within the last three treatment cycles. Whether this increase is associated with the stage of disease remains to be determined by kinetic studies in a larger group of patients.

In summary, we could demonstrate that 4 re-infusion cycles of *ex vivo *TKD/IL-2-activated PBMNC initiate and sustain an intrinsic NK cell-mediated cytolytic activity against autologous tumor and the NK cell target K562. This finding is in accordance to data derived from a clinical phase I trial [15] and could be confirmed in a pilot patient with malignant metastatic melanoma. An intrinsically enhanced cytolytic activity against Hsp70-positive tumor cells was observed in all patients who received more than 4 treatment cycles.

## Competing interests

The authors declare that they have no competing interests.

## Authors' contributions

GM contributed to conception and design, funding, supervision, data interpretation, writing and final approval of the manuscript. VM, SS, MG, BW, KH, and DM contributed to data collection and assembly of data. MM, WH, RI contributed to critical revision of the manuscript. All authors read and approved the final version of the manuscript.
